# Polyuria and polydipsia revealing a pituitary metastasis: Report of a rare case

**DOI:** 10.1002/ccr3.6025

**Published:** 2022-07-11

**Authors:** Ghassen Gader, Skander Guediche, Mohamed Ben Hadj Yahya, Mohamed Zouaghi, Mouna Rkhami, Mohamed Badri, Ihsèn Zammel

**Affiliations:** ^1^ Department of Neurosurgery Faculty of Medicine of Tunis Trauma and Burns Center University of Tunis‐El Manar Ben Arous Tunisia; ^2^ Department of Radiology Faculty of Medicine of Tunis National Institute of Neurology University of Tunis‐El Manar Tunis Tunisia

**Keywords:** metastases, pituitary, neurosurgery, neurooncology

## Abstract

Sellar region is a rare localization for intracranial metastases. The diagnosis is frequently delayed as symptoms are generally non specific. Radiologic diagnosis may be difficult as these tumours present similiraities to other more frequent sellar tumours. There is still no consensus regarding therapeutic approach. Prognosis is related to several features.

## Abstract 2

Pituitary metastases are rare tumors mainly diagnosed in patients with multiple metastatic lesions. Diagnosis is difficult as clinical symptoms are nonspecific. Treatment mainly aims to improve the quality of life.

## 1 INTRODUCTION

Sellar tumors are mainly represented by pituitary gland adenomas in adults and craniopharyngiomas in children. Pituitary metastases (PM) are among the less encountered tumors in this region, accounting for less than 1% of all brain metastases.^1^ PMs are not well‐documented lesions as most related publications are case reports,^2^, ^3^ but may be life threatening due to hormonal disturbances in severely compromised patients.^1^ Even in cancer patients, these conditions are frequently misdiagnosed as they do not always cause clinical disturbances, and are typically too small to be diagnosed on imaging.^4^ However, the number of documented pituitary metastases has increased probably due to the improvement of survival rates in cancer patients and the improvement of neuroradiology.^2^, ^5^ Here, we report the case of a patient suffering from a breast cancer, who was operated for a brain metastasis, and who presented postoperative polyuria and polydipsia related to a PM.

## 2 CASE REPORT

**FIGURE 1:**
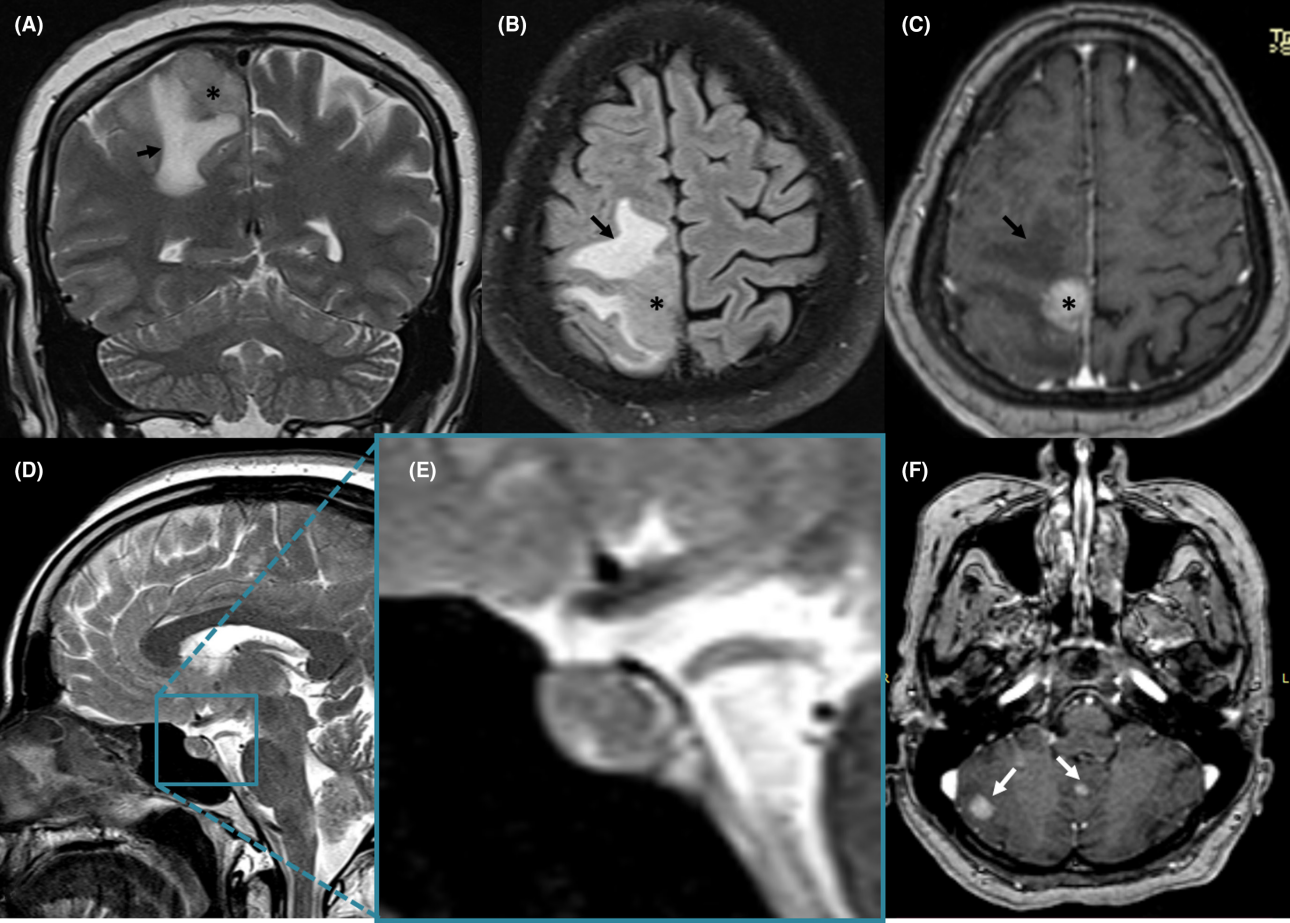
Parasagittal intra‐axial right rolandic mass lesion (white asterisk in “A–C”) presenting with T1, T2 (image A) and FLAIR (image B) isosignal and showing intense gadolinium enhancement (image C). Extensive fronto‐parietal vasogenic edema is seen around the lesion (black arrows in “A–C”). Images “D” and “E” show a normal hypophyse with no supra‐sellar lesion. Note the subtentorial metastatic lesions in the right cerebellum and the vermis (white arrows in “F”) presenting as round lesions with intense gadolinium enhancement

**FIGURE 2:**
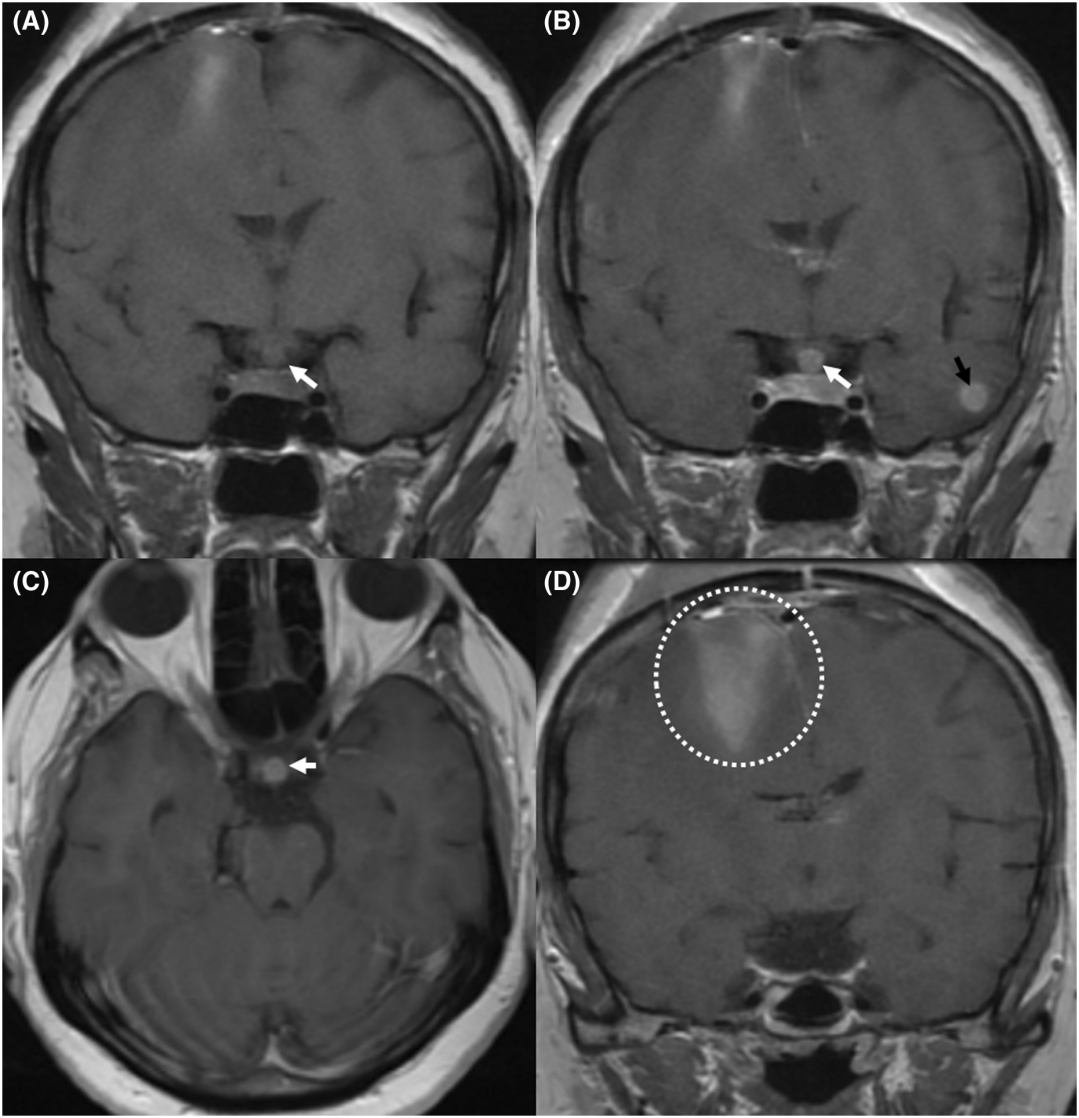
Postoperative MRI screening shows a new lesion centered on the pituitary stalk presenting as a T1 isointense round mass (white arrow in “A”) with homogeneous enhancement after gadolinium injection (white arrows in “B” and “C”). Note the left temporal lobe intra‐axial metastasis (black arrow in “B”) and the parenchymal enhancement of the operatory site (dotted circle in “D”)

We report the case of a 54‐year‐old woman, who was diagnosed with a right breast cancer in 2019. She underwent at first a radical mastectomy. Pathologic examination concluded to a ductal carcinoma. Afterward, she had radiotherapy and chemotherapy. The patient was considered to be in remission in November 2020 as investigations showed neither local recurrence nor any metastatic localizations. In November 2021, the patient presented the progressive onset of a numbness in her left lower limb, as well as headaches without vomiting or visual blur. Physical examination found a paresis in the left lower limb, with no other neurologic signs of localization. A brain MRI was performed (Figure 1), showing three intracranial lesions: two nodular in the posterior fossa and one supratentorial rolandic parasagittal lesion. The rolandic lesion was obviously responsible for the motor deficit, thus the decision to operate the patient for resection. Preoperatively, the tumor was infiltrating, hemorrhagic, in contact with the lateral wall of the superior sagittal sinus. A complete resection was performed. Postoperatively, the patient presented a worsening of her motor status as she developed a left hemiparesia that regressed after 2 days of corticosteroid therapy. Otherwise, she presented on the third postoperative day a polyuria quantified 8 liters per day, associated with a polydipsia. Ionogram showed a hypernatremia of 155 mmoL/L. These elements were concordant with a diabetes insipidus, and as this complication could not be related to surgical manipulation, a postoperative MRI (Figure 2) was performed. This examination showed two new lesions that were not present on the preoperative MRI (performed 1 month before surgery): The first was left temporal, and the second was centered on the pituitary stalk. The patient received DESMOPRESSINE, leading to a regression of the diabetes insipidus. The patient was referred for adjuvant radiotherapy.

## 3 DISCUSSION

Pituitary metastases (PM) are among the rarest sellar tumors as they represent 1.8% of all pituitary masses^2^ and 0.4%–3.6% of all brain metastases.^1^, ^3^ PMs can be a secondary localization for every cancer, but lung, breast, digestive, renal, hematologic neoplasms and prostate are more often the source for these metastases.^6^ Breast cancer is the most frequent primitive tumor for PMs, as such patients are 9.3 times more subject to present these metastatic localizations.^7^ PMs are most often diagnosed in adult patients, mainly during the sixth decade, without any gender predominance.^8^ When diagnosed, PMs are frequently associated with multiple metastatic sites.^1^ PMs are frequently diagnosed postmortem, as symptoms are usually nonspecific or absent, which explains the delayed diagnosis in patients with limited survival rate. Symptoms can be nonspecific such as headaches and asthenia, or be related to hormonal dysfunction varying from diabetes insipidus to other signs related to endocrinal disturbance. The most encountered abnormality is a high Prolactin level which usually remain less than 200 ng/ml, related to stalk compression.^9^ Hypogonadism, hypocorticism, and hypothyroidism have been reported in very few cases.^1^

Radiologic features of PMs on MRI are usually source for difficulty in differentiation with other more frequent sellar tumors, mainly pituitary adenomas. PMs are frequently non‐homogenous invasive sellar masses, associated with a loss of the posthypophyse bright spot and an erosion without any enlargement of the sella.^10^ When PMs are isolated without any possibility of radiologic differentiation, the evolution of the tumor may be helpful as a rapid increase of a sellar mass with aggressive infiltration of surrounding tissues is the argument for PMs.^11^ Another argument is that the infundibular recess invasion by a suprasellar tumor is in favor of PMs, contrary to adenomas with suprasellar expansion which the mainly extend posteriorly.^12^ Moreover, an enhancement of the infundibulum is rather related to PMs rather than to adenomas.^11^, ^12^

In cancer patients, the differentiation between metastasis and benign lesion is not only essential for the therapeutic plan, but also to avoid unnecessary surgery.^4^, ^9^ There is no therapeutic consensus regarding PMs. Surgery may be proposed for patients in good conditions, allowing to confirm the diagnosis and to decrease symptoms. However, the frequent invasion of the cavernous sinus and the hypervascularization of the tumor make total resection difficult.^13^ Radiotherapy, radiosurgery, and intrathecal chemotherapy have also been proposed with variable results, but their reliability has hardly been hindered by a frequent and severe post‐therapeutic panhypopituitarism.^2^

Prognosis and survival rate are related to the presence of other metastases, and the subtype of the primitive tumor. The mean survival rate varies between 6 and 13.6 months.^2^, ^14^, ^15^ These differences may be related to the timing of diagnosis, the general condition, and the nature of the proposed treatment.

## 4 CONCLUSIONS

Despite their rarity, PM should be diagnosed as early as possible to allow an appropriate treatment, to optimize the quality of life, and to insure the longest possible survival time.

## AUTHOR CONTRIBUTIONS

GG wrote the manuscript. SG, MHY, and MZ collected documentations. MR made the bibliographic research. MB and IZ corrected the manuscript.

## CONFLICT OF INTEREST

The authors declare having no conflicts of interest regarding this paper.

## CONSENT

We have a written consent from the patient regarding this paper.

## Data Availability

None.
